# Relation Between Fracture Stability and Gas Leakage into Deep Aquifers in the North Perth Basin in Western Australia

**DOI:** 10.1111/gwat.12853

**Published:** 2019-02-25

**Authors:** F. Mullen, H. Boogaerdt, R. Archer

**Affiliations:** ^1^ Department of Engineering Science The University of Auckland Private Bag 92019, Auckland Mail Centre, Auckland 1142 New Zealand

## Abstract

The Kockatea Shale is a proposed target for unconventional gas development in the North Perth Basin in Western Australia. This research is concerned with correlating the extent of thermogenic gas leakage into deep aquifers overlying the Kockatea Shale with an assessment of how close the formation is to mechanical failure. Data from two petroleum exploration wells located approximately 20 km apart were considered. Both have comparable stratigraphy; however, they differ by their local tectonic setting. The stress regime is strike slip at Arrowsmith 2 well and for an assumed hydrostatic pressure the Kockatea Shale is not close to frictional limits. Minor amounts of methane and trace amounts of short chain alkanes are leaking into deep aquifers pre‐development. In contrast, the stress regime is strike slip/normal at Woodada Deep well and the Kockatea Shale is close to frictional limits. Significant volumes of gas including methane and condensate are leaking into deep aquifers. The sealing capacity of the Kockatea Shale as evidenced by the variation in gas concentration in aquifers at the two sites indicates the formation is sensitive to stress. Additionally given the low permeability of the regional Kockatea Shale seal, it is assumed that at both locations gas leakage is via critically stressed faults. Deep aquifers proximal to the shale gas target are low salinity (<5000 ppm NaCl eq.) at Woodada Deep well and are saline at Arrowsmith 2 well. Based on this assessment, it is suggested that hydraulic fracture stimulation at the Woodada Deep well poses a significant environmental risk.

## Introduction

Despite the controversy surrounding the potential for leakage of gas and other fluids during hydraulic fracturing, there is no requirement in the regulatory guidelines for unconventional gas companies to assess seal integrity in unconventional reservoirs. (Department of Mines and Petroleum Western Australia 2015). This research is concerned with the estimation of the principal stress components for two exploration wells in the North Perth Basin. The goal of the study is to relate these principal stress estimates to estimates of how close the stratigraphic sections under consideration are to mechanical failure. Conclusions are drawn from this on potential pathways for gas leakage from shale gas reservoirs into deep (groundwater) aquifers in overlying formations. The methodology described provides regulators with a screening level risk assessment suitable for appraising the potential subsurface impacts of hydraulic fracturing. At the present time, risk assessments associated with hydraulic fracturing are restricted to numerical modeling and the use of microseismic and these are discussed below.

Rutqvist et al. (2015) used a coupled multiphase fluid flow and geomechanical simulator to model slip events in the context of hydraulic fracturing. The fault rupture zone extended a maximum of 200 m from the injection well. However, the permeability of the fault zone was 10^−19^ m^2^ and was the same as for unfaulted rock. Faults are usually formed by a fault core and a damage zone on both sides of the core. Damage zones are usually more permeable than the host rock (Cappa and Rutqvist 2011). In addition, permeability in faults may be pressure‐dependent (Finkbeiner et al. 2001; Chen et al. 2013). Vertical extent of microseismicity has generally been used to confirm that overlying aquifers are not affected by hydraulic fracturing operations (Flewelling et al. 2013). However, the rate of growth of hydraulic fractures within shale is insufficient to generate seismically detected signals (Rutledge 2009). Mass balance analysis of microseismic deformation indicates this only accounts for a small fraction of the volumetric deformation implied by production volumes from shale gas reservoirs (Warpinski et al. 2012) and suggests slow aseismic slip in shale makes a significant contribution to production (Kohli and Zoback 2013). Hydrocarbon leakage from shale seals can occur along faults that are ideally oriented for failure in the in situ stress field (Dewhurst et al. 2004).

This research is concerned with the pre‐development geomechanical stress in a shale gas target. The stress in the Earth's crust can be described by the principal stresses, S1, S2, and S3. The principal stresses are the normal stresses. Within the lithosphere the principle planes appear to be oriented approximately horizontally and vertically (Anderson 1951). *S*_*v*_ describes the vertical stress, *S*_*H*max_ describes the maximum principal horizontal stress and *S*_*h*min_ the minimum principal horizontal stress. Three stress regimes and the relative magnitude of the principal stresses are shown in Table 1.

**Table 1:** Possible Stress States in the Earth's Crust (Anderson 1951)

Stress Regime	Magnitude of the Principal Stress
Normal	*S*_*v*_ > *S*_*H*max_ > *S*_*h*min_
Strike slip	*S*_*H*max_ > *S*_*v*_ > *S*_*h*min_
Reverse	*S*_*H*max_ > *S*_*h*min_ > *S*_*v*_

Published estimates of stress at depths of greater than 1000 m indicate the stress is strike slip (Le and Rasouli 2012) becoming normal at increasing depths (Rasouli and Sutherland 2014) in the North Perth Basin. These estimates are not considered reliable because the horizontal stresses were based on empirically determined Poissons ratios derived from sonic logs. While these estimates may have some local usefulness, such methods have little or no predictive value. If carefully calibrated stress data is locally available then data from sonic logs can be used to extend the analysis to nearby wells however, the method cannot be used in isolation in a frontier well (Zoback 2007).

In this work, the stress state is estimated for the Kockatea Shale at Arrowsmith 2 well (2300 m depth) and Woodada Deep well (1910 m depth). The horizontal stresses derived by credible means are plotted on stress diagrams to determine how close the Earth's crust is to frictional limits. The stress data are then correlated with gas leakage data acquired from well completion reports.

## Location

**Figure 1:**
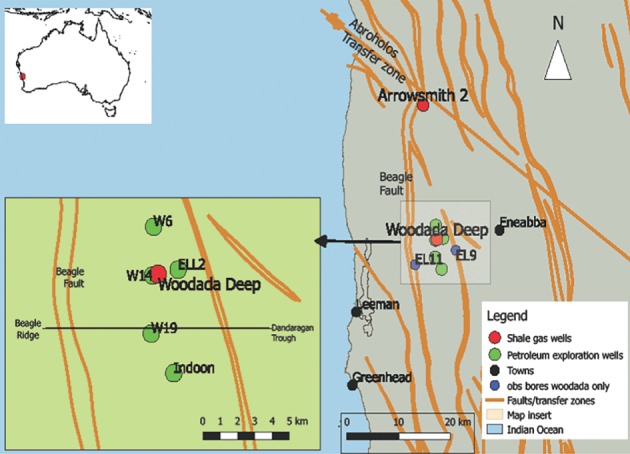
Location map of Arrowsmith 2 and Woodada Deep shale gas wells in red, old petroleum exploration wells in green and groundwater observation bores in blue. Structural features include the Abroholos Transfer Zone and the Beagle Fault. The map insert shows relevant wells in the Woodada gasfield and the approximate location of the cross section from Beagle Ridge to the Dandaragan Trough shown in Figure 2.

The locations of the new shale gas exploration wells are shown in Figure 1. The Arrowsmith 2 well and the Woodada Deep well are approximately 20 km apart in an agricultural area in the mid‐west of Western Australia.

## Geology

The Perth Basin is an elongated rift basin and covers approximately 100,000 km^2^ along the south west coast of Australia. The Cadda Terrace in the North Perth Basin is the focus of this study. The Terrace is an area of uplift bounded by the Beagle Ridge to the west and the Dandaragan Trough to the east (Figure 1). The northern boundary is defined by the Abrolhos Transfer zone. The southern boundary is poorly defined as it is covered by post breakup Cretaceous rocks in an area of poor seismic resolution (Mory and Iasky 1996).

**Figure 2:**
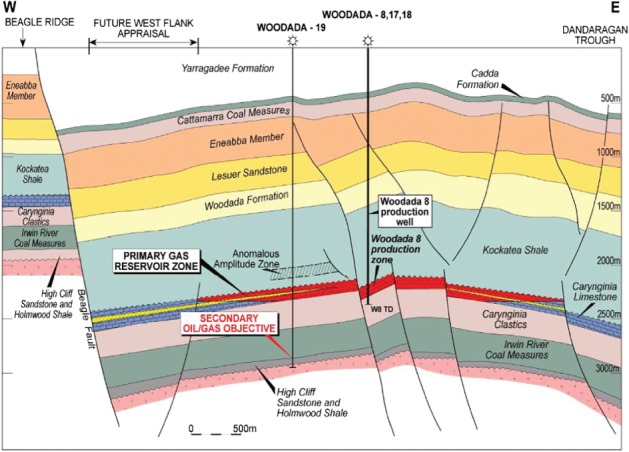
Schematic east‐west cross section of Woodada gas field showing the Beagle Fault (Hardman Resources 2003). Location of cross section can be seen in Figure 1.

The Woodada Deep well (formerly known as the Woodada 4 well) is located on the Woodada structure which is a north plunging faulted anticline. An east‐west cross section is shown in Figure 2. Faulting extends into the Eneabba Formation based on seismic surveys (Hydrocarbon Resource Group 1996).

The Arrowsmith 2 well is located near the intersection of the north trending Beagle Fault system and the Abrolhos Transfer Zone which defines the northern edge of the Cadda Terrace (Figure 1). The throw of the Beagle Fault system ranges from 500 to 1200 m and the fault extends to the surface (Mory and Iasky 1996). No continuous fault plane is associated with the Abroholos Transfer Zone; however, it is identified by swings and terminations in major north trending faults (Song et al. 2001).

The stratigraphy for the Woodada area is shown in Figure 2 and is similar to the Arrowsmith 2 area however the Kockatea shale is 1572 m below ground at Woodada Deep and 2226 m below ground at Arrowsmith 2 reflecting a regional dip to the north. The Kockatea Shale is a major oil source rock and seal in the Perth Basin (Mory and Iasky 1996). Petroleum resource estimates indicate the formation may have up to 8 trillion cubic feet (Tcf) of gas and 500 million barrels of oil/condensate in the North Perth Basin (Kuuskraa et al. 2013). The formation below the Kockatea Shale is the Carynginia Limestone in the Woodada gasfield and Wagina Sandstone at Arrowsmith 2. The Carynginia Limestone was a conventional gas target in the 1980s; however, it is not defined in well completion reports.

## Hydrogeology

In Western Australia, deep aquifers (>300 m) are likely to become an increasingly important water resource in the context of climate change. Deep aquifers are often saline however those with salinities of less than 5000 ppm NaCl are used for livestock (Department of Primary Industries and Regional Development 2018). The Lesueur Aquifer is the deepest useful aquifer in the study area. It outcrops at a groundwater divide located approximately 40 km south of Woodada Deep well and then dips northwards. At this location the aquifer has a salinity of <500 ppm and recharge as a percentage of rainfall may be as high as 5.6% (Commander 1978). Prevailing flow is northwards due to a no flow boundary created by the north trending Beagle Fault (Commander 1978). At the Woodada Deep well, the top of the Lesueur Aquifer is 1186 m deep and water is marginal to brackish based on resistivity log estimates (Mullen 2017, unpublished manuscript). The deep potentiometric data show no seasonal or annual fluctuations. Discharge is via upward leakage along the Beagle Fault zone (Commander 1978). The overlying Eneabba Formation has a salinity of 600 to 1920 ppm based on water samples from the EL11 and EL9 monitoring bores (Figure 1). Both aquifers are NaCl dominated and are assumed to be hydraulically connected (Commander 1978). Groundwater levels are declining in the shallow aquifers (Department of Water 2011) and the Lesueur Aquifer is likely to become increasingly important for agricultural water supply in this area. At the Arrowsmith 2 well, the top of the Lesueur Aquifer is 1810 m deep and water has a salinity of 75,000 ppm NaCl equivalent based on drill stem tests conducted in the adjacent Arrowsmith 1 well (West Australian Petroleum 1965).

## Research Method

The objective of this research is to assess the correlation between stress in the Kockatea Shale and gas concentration in the overlying aquifers. In this section, the methods used to derive inputs for stress limit diagrams are discussed. These include the vertical and horizontal stress, rock strength, pore pressure, and borehole pressure. The assumptions used in the interpretation of the gas concentration in aquifers data are briefly discussed.

The vertical stress (*S*_*v*_) is directly calculated from the density log as the integration of density of different formation layers (Equation 1)

(1) $$\mathrm{Sv}∼\int_{0}^{z}\rho \left(z\right)g\;\mathrm{dz},$$

where *z* is the depth (m), *ρ* is the density of the overlying sediment (g/cm^3^), and *g* is the acceleration due to gravity (m/s^2^). Data was screened for washouts using the density correction log (DHRO). If DHRO was greater than 0.05 then the density log estimates were removed. A power law function was fitted to smooth the *S*_*v*_ data (*S*_*v*_ = 0.0131*depth (m)^1.0852^).

At Arrowsmith 2 well the density log was restricted to the shale gas targets; however, a sonic log was available at the nearby Arrowsmith 1 well. This well is located 300 m north of Arrowsmith 2 and it is assumed that the stress conditions are constant across this distance. An empirical formula from Brocher (2005) for the Nafe‐Drake graphical relationship (Ludwig et al. 1970) between density and compressional wave velocity was used (Equation 2) and is as follows:

(2) $$\begin{array}{cc} \rho \left(g/{\mathrm{cm}}^{3}\right) & =\left(1.6612^{*}V_{p}\right)-\left(0.4721{V_{p}}^{2}\right)\\ & \;+\left(0.0671{V_{p}}^{3}\right)-\left(0.0043{V_{p}}^{4}\right)+\left(0.000106{V_{p}}^{5}\right), \end{array}$$

where *ρ* is density and *V*_*p*_ is compression wave velocity. This equation can be used in the *V*_*p*_ range 1.5 to 8.5 km/s. The *V*_*p*_ range for the Arrowsmith 2 well ranges from approximately 1.5 km/s near surface to 3.7 km/s at the base of the Kockatea Shale at a depth of 2691 m. A bulk density of 1.4 g/cm^3^ was assumed from the surface to a depth of 53 m to account for missing data. The density data from Arrowsmith 2 well was combined with sonic derived density data from the Arrowsmith 1 well. The resulting *S*_*v*_ estimate was smoothed using a linear function (*S*_*v*_ = 0.0262*depth(m) − 2.0014). The regression was used to estimate *S*_*v*_ for the depth of interest in the Kockatea shale (2300 m).

The magnitude of the minimum horizontal stress (*S*_*h*min_) can be estimated from leak off tests (LOTs). During the test, the pressure is increased until a fracture has formed at the borehole wall. Fracture formation is marked by a change in slope on a pressure versus time plot (Fjaer et al. 2008). There were no LOT data for the Woodada Deep well. The Woodada 6 well to the north of Woodada Deep and Indoon well to the south are on the same north to south structural trend as Woodada Deep (Figure 1). The *S*_*h*min_ gradients at these two wells were assumed to be representative for the Woodada Deep well. Using the combined LOTs the predicted *S*_*h*min_ was interpolated from the following relationship: *S*_*h*min_ = (0.0147*depth(m) + 2.0056).

At Arrowsmith 2 the *S*_*h*min_ was estimated using LOTs conducted in the Cadda Formation at a depth of 603 m depth and Kockatea Shale at a depth of 2281 m. The *S*_*h*min_ was interpolated from the following relationship: *S*_*h*min_ = (0.0181*depth(m) + 0.2115).

Pore pressure is assumed to be hydrostatic in the Kockatea Shale on the Cadda Terrace based on the work of Ahmad et al. (2014) using Eatons method (Eaton 1975). This method can only predict shale pore pressure reliably if the rocks compaction history has exclusively followed a loading trend (Finkbeiner et al. 2001). Comparison of the sonic velocity with resistivity and density logs provide a qualitative indication of overpressure where those logs existed. If sonic and resistivity undergo reversals but the density log does not then this suggests overpressure (Bowers 2001). There was evidence of overpressure in some intervals at Woodada 1, Woodada 6, and Indoon wells (data not shown). No assessment could be made for Woodada Deep or Arrowsmith 2 wells due to issues with geophysical log data. Pore pressure was assumed to be hydrostatic in both Woodada Deep and Arrowsmith 2 given both wells are located on the Cadda Terrace, however, overpressure is possible in some intervals based on the above assessment.

Borehole pressure is required for *S*_*H*max_ estimation. Borehole pressure during drilling is referred to as Δ*P*_*w*_. Excess pressure in the borehole equates to a positive Δ*P*_*w*_ and a minus Δ*P*_*w*_ means there is excess pressure in the formation. Based on the Woodada 4 well completion report, deviation problems were encountered below a depth of 1814 m and mud weight was increased to 1.13 g/cm^3^ which equates to 20.1 MPa. Hydrostatic pressure at this depth is 18.1 MPa so the difference between the pressure in the mud and the pressure in formation is assumed to be 2 MPa at the target depth (1910 m). At Arrowsmith 2 well a linear interpolation of the difference between mud pressure and hydrostatic pressure is approximately 3.7 MPa at a depth of 2300 m.

Unconfined compressive strength (UCS) is required for the maximum horizontal stress estimate. In the absence of rock strength data at the Woodada Deep and Arrowsmith wells, UCS was estimated using the following relationship from Lashkaripour and Dusseault (1993):

(3) $$\mathrm{UCS}=1.001\emptyset^{-1.143},$$

where ∅ is the porosity and UCS is in MPa. This relationship is suitable for low porosity (<10%) high strength (∼79 MPa) shales. The equation fits 90% of available data within ±10 MPa (Chang et al. 2006). Porosity can be determined from the density log (Smithson 2012). The equation was tested for the Redback 2 well located 30 km north of the Woodada gasfield where laboratory derived strength data exists for the Kockatea Shale. The laboratory results indicate a mean UCS of 92 MPa for the depth interval 3788 to 3835 m (Origin Energy 2010). Using Equation 3, and an average grain density of 2.68 g/cm^3^ from the Redback 2 core, the log derived geometric mean strength for the same depth interval was 100 MPa. This estimate is considered comparable to the laboratory derived estimate.

Using the same grain density (2.68 g/cm^3^) for the porosity estimate, the average log derived UCS was estimated to be 32 MPa at Woodada Deep well for the depth interval 1740 to 1925 m. This is considerably lower than the log derived estimate from the Redback 2 well (92 MPa). However, the estimates for the Redback 2 well are for shale intervals buried at significantly greater depths compared with the Woodada Deep well. Burial induced diagenetic changes in clay minerals can result in increased strength in shale formations (Dewhurst et al. 2004). The UCS was estimated to be 49 MPa for the Arrowsmith 2 well using Equation 3 and grain density of 2.74 g/cm^3^ from drill core (Norwest Energy 2011).

The estimate of the maximum horizontal stress (*S*_*H*max_) can be constrained using Equation (4) (Barton et al. 1988):

(4) $$S_{H\max}=\frac{\left(\mathrm{UCS}+\Delta \mathrm{Pw}+2\mathrm{Pp}\right)}{\left(1-2\cos2\theta \right)}-S_{h\min}\frac{\left(1+2\cos2\theta \right)}{\left(1-2\cos2\theta \right)},$$

where UCS is the unconfined compressive strength, Θ is the distance from the edge of breakouts from *S*_*H*max_ (67.5° for an 8 in. hole), Δ*P*_*w*_ is excess pressure in the well (negative if drilled underbalanced) and *P*_*p*_ is pore pressure. This equation requires that breakouts are confirmed to exist in the borehole. Breakouts are damage zones oriented towards the minimum horizontal stress in well bores and are related to hoop or circumferential stresses. The minimum circumferential stress occurs at *S*_*H*max_ and the maximum circumferential stress occurs at *S*_*h*min_ (Barton et al. 1988). Published breakouts indicate an *S*_*H*max_ direction of 110°N at Woodada Deep (Reynolds et al. 2006). Breakouts are documented for the Arrowsmith 2 well at 101°N (Norwest Energy 2011).

For a specified depth, Equations 5 and 6 define limits for the occurrence of borehole breakouts (Grandi et al. 2002) and further constrain *S*_*H*max,_

(5) $$S_{H\max}=\left({\mathrm{Co}}_{\left(\sigma 90\right)}+S_{h\min}+P_{p}+\Delta P_{w}\right)/3,$$

where C_o(*σ*90)_ is the rock strength when breakouts are restricted to *S*_*h*min_.

(6) $$S_{h\min}=\left({\mathrm{Co}}_{\left(\sigma 0\right)}+S_{H\max}+P_{p}-\Delta P_{w}\right)/3,$$

where Co_(*σ*0)_ is the rock strength when breakouts cannot occur.

Stress can range from lithostatic in the absence of tectonic forces to the limit defined in Equation (7) (Zoback and Healy 1984):

(7) $$\frac{S1-\mathrm{Pp}}{S3-\mathrm{Pp}}\leq 3.1,$$

where *Pp* is the pore pressure, S1 is the maximum principal stress, and S3 is the minimum principal stress. This equation is used to generate the stress limit diagram and assumes fault rock has a coefficient of friction of 0.6 and no cohesion (Zoback and Healy 1984). Horizontal stress data that plots near the circumference of the stress limit diagram corresponds to a case of active faulting (Zoback et al. 2003).

Finally, gas levels and type are logged during drilling of exploration wells and this data is used in conjunction with the stress limit diagrams to assess the relationship between gas concentrations in overlying aquifers and the occurrence of mechanical failure in the gas target. Gas concentration data logged during drilling can be used with some caveats to infer its origin. If methane levels in aquifers follow the same trends as condensate (ethane, butane, pentane, etc) then it is assumed the gas has a common thermogenic origin. The Kockatea Shale produces a wet gas comprising methane −73.2%, ethane −12.2%, propane −10.8%, butane −3%, pentane −0.8%. These ratios remain constant with depth indicating that this is an oil prone formation (Sagasco Resources 1993).

## Results

In this section, the stress data are plotted on stress limit diagrams for the Arrowsmith 2 and Woodada Deep wells. Gas concentration data is also presented graphically for overlying aquifers at each site. Stress data for both wells are summarized in Table 2. The stress diagrams comprise three triangles. The smallest circumscribes normal faulting, the intermediate triangle, strike slip faulting and the largest defines reverse faulting. An increase in pore pressure above hydrostatic will reduce the size of the polygon making active faulting more likely.

**Table 2:** Summary of Inputs for Stress Diagrams

Well	Depth (m)	*Pp* (MPa)	Δ*Pw* (MPa)	*S*_*v*_ (MPa)	UCS (MPa)	*S*_*h*min_ (MPa)	*S*_*H*max_ (MPa)
Woodada Deep	1910	18.7	2	47.6	27–32	30.1	45.8–50.8
Arrowsmith 2	2300	22.5	3.7	57.7	38–49	41.8	62–73

## Woodada Deep Well

**Figure 3:**
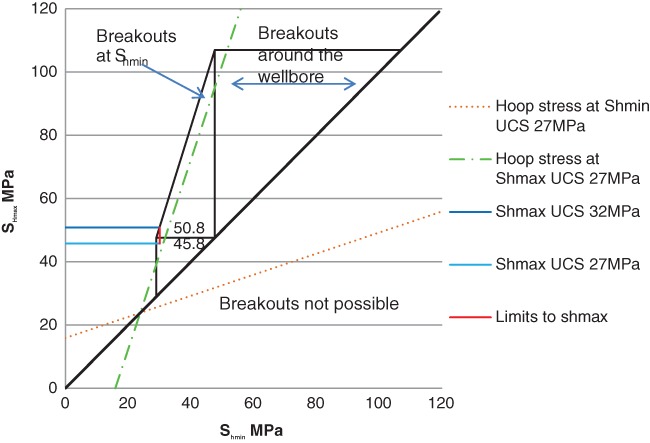
Stress limit diagram of the Woodada Deep well. This defines the possible magnitudes of *S*_*h*min_ and *S*_*H*max_ at a depth of 1910 m as defined by Anderson faulting theory and Coulomb faulting theory for a given coefficient of friction (*μ* = 0.6) and hydrostatic pore pressure. A UCS of 32 MPa results in an upper limit of *S*_*H*max_ of 51 MPa (dark blue line). A UCS of 27 MPa results in a lower limit of *S*_*H*max_ of 46 MPa (light blue line). Breakouts will be restricted to *S*_*h*min_ if the horizontal stress plots to the left of the green dotted line. The stress state is such that breakouts will always occur for this stress regime as the data plots above the brown dotted line.

At Woodada Deep well the stress regime plots across both strike slip and normal faulting zones (Figure 3). The red line defines the range of allowable *S*_*H*max_ (45.8 to 50.8 MPa) for the inputs defined in Table 2, a coefficient of friction of 0.6 and hydrostatic pore pressure. Based on these inputs the Earth's crust is close to frictional limits.

**Figure 4:**
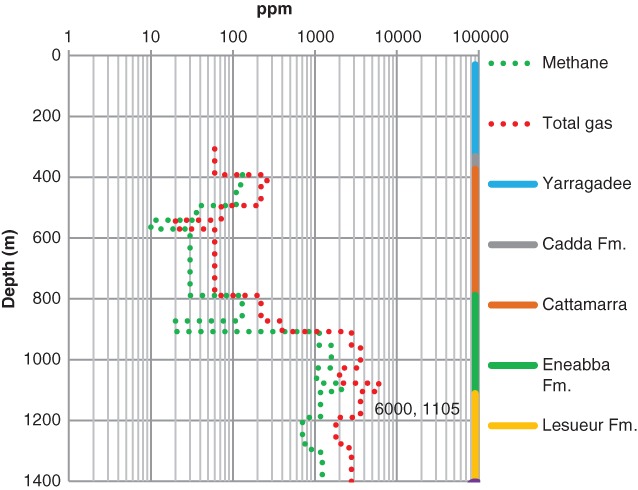
Woodada 14 well recordings of gas levels start at 307 m depth. Total gas (red dotted line) is contrasted with methane (green dotted line). Aquifers are color coded on the right axis. A maximum total gas of 6000 ppm was recorded at 1105 m depth.

There is no gas data for Woodada Deep; however, gas data for the Woodada 14 well located 300 m WSW from Woodada Deep (Figure 1) is shown in Figure 4. Gas data was logged from 307 m depth. There are large volumes of both methane and condensate in the Lesueur Aquifer (maximum of 6000 ppm at 1105 m depth). The trends in methane match the trends in total gas suggesting the gas is from a common thermogenic source. The Lesueur Aquifer and Eneabba Formation are functioning as gas collector zones.

## Arrowsmith 2 Well

**Figure 5:**
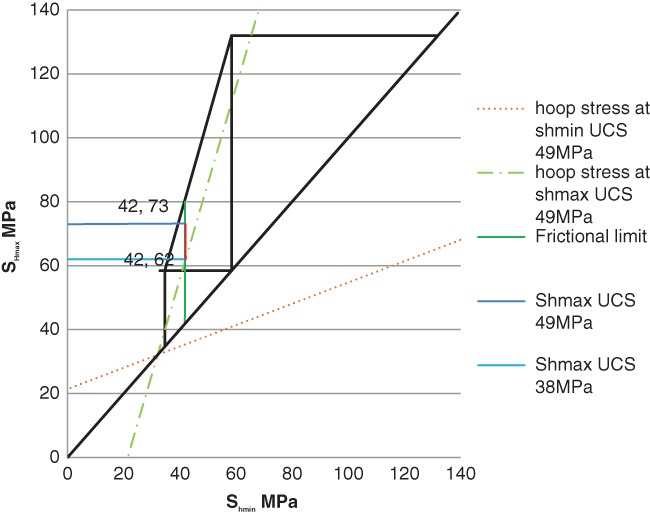
Stress diagram Arrowsmith 2. This defines the possible magnitudes of *S*_*h*min_ and *S*_*h*max_ at 2300 m depth as defined by Anderson faulting theory and Coulomb faulting theory for a coefficient of friction of 0.6 and hydrostatic pore pressure. *S*_*H*max_ can vary between 62 and 73 MPa.

**Figure 6:**
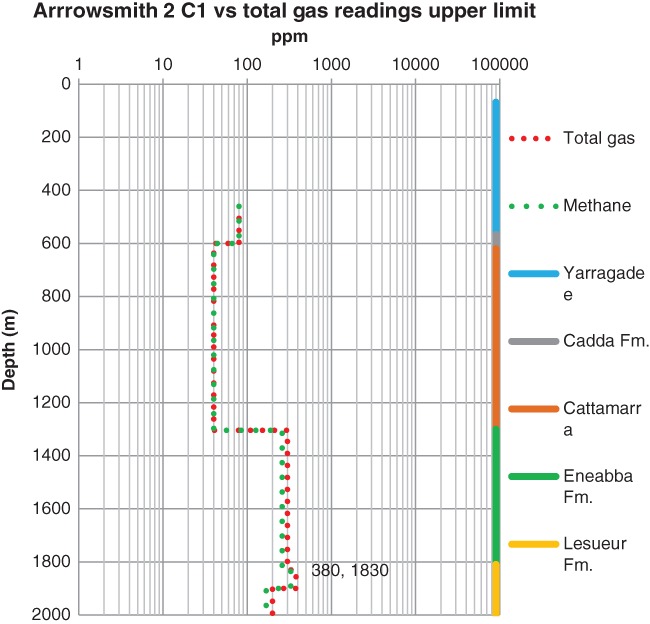
Arrowsmith 2 well gas data start at 460 m depth. Formations are color coded on the right axis. Maximum total gas is 380 ppm at 1830 m depth in the Lesueur Aquifer.

For the Arrowsmith 2 well, the stress state is strike slip and not close to frictional limits for a coefficient of friction of 0.6 and hydrostatic pressure (Figure 5). *S*_*H*max_ may vary between 62 and 73 MPa. At the Arrowsmith 2 well the gas recordings begin at 460 m depth. In the deep aquifers, gas is primarily methane (Figure 6). Total gas reaches a maximum concentration of 380 ppm at 1830 m depth in the Lesueur Aquifer. The Eneabba Formation and Lesueur Aquifer are gas collector zones.

## Discussion

The Earth's crust is close to frictional limits in the Woodada area whereas this is not the case at the Arrowsmith 2 well. Significantly, gas leakage is an order of magnitude higher into the Lesueur Aquifer in the Woodada area compared with the Arrowsmith 2 well. The data suggests the extent of hydrocarbon leakage into aquifers can be correlated with the stress state in the Kockatea Shale in the North Perth Basin. These results have implications for the way shale gas developments are assessed in terms of their environmental impact.

Hydrocarbon leakage may occur as a result of fault juxtaposition issues. These refer to the creation of connected carrier beds via juxtaposition of different permeable formations/layers across faults. Fault juxtaposition is not considered to be a significant issue in the Woodada gasfield as throw on the bounding faults is generally less than the thickness of the Kockatea Shale. In addition permeability is assumed to be low in the Kockatea Shale. The occurrence of the Carynginia Limestone as a conventional gas target beneath the Kockatea Shale indicates the formation functions as a seal in this location. Buoyancy alone is not likely to be a significant mechanism for gas leakage at Woodada gasfield.

Methane in the Lesueur Aquifer at the Arrowsmith 2 well could potentially migrate up dip to the Woodada gas field 20 km to the south. Whether this could contribute to the higher gas concentrations seen in the Lesueur Aquifer at Woodada gasfield is difficult to determine. However, gas type varies across the Woodada structure in the Lesueur Aquifer based on the orientation of the fault system. Fracture stability analysis indicated faults oriented NW, are critically oriented for shear failure in the current stress field even at hydrostatic pressure (Mullen 2017, unpublished manuscript). Wells adjacent to these faults are more likely to have significant concentrations of condensate in the Lesueur Aquifer (e.g., Woodada 14 well). Concentrations vary with depth implying limited mixing has occurred. In contrast, wells adjacent to faults which are not critically oriented for shear failure (oriented NE) are more likely to have methane only in adjacent petroleum wells, for example, ELL2 well. In this case gas concentration is constant with depth suggesting mixing has occurred due to lateral spread. This indicates that while it is difficult to speculate about the origin of methane due to its relative mobility, the distribution of heavier long chain hydrocarbons is likely to reflect a local source. It is argued on this basis that the elevated gas levels in the Lesueur Aquifer at Woodada Deep well are controlled locally by faults and are not the result of up dip migration in aquifers from Arrowsmth 2 well towards Woodada gasfield.

A difference in maturity or total organic carbon content in the Kockatea Shale could potentially account for higher gas concentrations at Woodada. The total organic carbon content is 0.6 at Arrowsmith 1 well and 0.5 at Woodada 6 in the Woodada gasfield (Tupper 1992) indicating comparable generative potential at both locations. In addition, both gas and oil were produced during hydraulic fracturing of the Kockatea Shale at Arrowsmith 2 (Norwest 2012).

Variations in the thickness of the Woodada Formation (located between the Lesueur Aquifer and the Kockatea Shale) could potentially account for some of the differences in gas concentration at the two sites. The Woodada Formation is 40 m thicker at Arrowsmith 2 compared with Woodada Deep. However the Woodada Formation is an aquifer and is not considered to be a reliable seal in the context of methane migration from the Kockatea Shale into the Lesueur Aquifer.

Episodic flow of hydrocarbons into shallow strata via ruptured faults in a fault valve fashion is possible (Finkbeiner et al. 2001; Chen et al. 2013). For this mechanism to operate, pore pressures are required to be as high as the least principle stress in the overlying strata (Nur and Walder 1990). While pore pressure is assumed to be hydrostatic in the Kockatea Shale there is evidence of overpressure in some intervals of the Kockatea Shale based on trends in sonic, density and resistivity logs in the Woodada gasfield. Fault reactivation and/or naturally occurring hydraulic fracturing in faults may be contributing to gas leakage in this area.

There are uncertainties in the geomechanical model. Specifically hydrostatic pore pressure cannot be assumed for all intervals of the Kockatea Shale. In addition the limit diagrams are based on an assumption of cohesionless rock and a coefficient of friction of 0.6 (Byerlee 1978). Dynamic poroelastic effects (stress‐pore pressure coupling) will reduce the likelihood of failure; however, this requires numerical modeling (Yielding personal communication, 2019). Due to the uncertainty in geomechanical models, van Ruth et al. (2006) suggests that fault reactivation analysis cannot be used directly to measure the maximum allowable pore pressure increase a fault can withstand prior to reactivation. Monte Carlo simulations have been used to assess the uncertainty in geomechanical parameters in the context of CO_2_ sequestration (Hung and Wu 2012). This approach may have some utility in assessing risk to groundwater from hydraulic fracturing.

## Conclusion

Stress analysis based on various parameters acquired during drilling suggests that faults in the Woodada Gas Field are critically stressed and are likely to be conductive to gas. This is corroborated by naturally high levels of gas present in aquifers above the Kockatea Shale source rock. There is potential for movement along critically stressed faults during hydraulic fracture stimulation, with consequent increase of gas leakage to overlying aquifers. A screening level assessment based on stress analysis using data acquired from oil exploration wells is a useful application to determine the level of risk to overlying aquifers from increased gas leakage resulting from hydraulic fracture stimulation.

## Authors' Note

The authors do not have any conflicts of interest or financial disclosures to report.
